# Using Best Programs for Caregiving to Access Evidence-Based Dementia Care

**DOI:** 10.1093/geroni/igaf122.829

**Published:** 2025-12-31

**Authors:** Morgan Minyo, David Bass, Rachel Cannon, Zoe Fete, Megan Huth, Sara Powers

**Affiliations:** Benjamin Rose Institute on Aging, Cleveland, Ohio, United States; Benjamin Rose Institute on Aging, Cleveland, Ohio, United States; Benjamin Rose Institute on Aging, Cleveland, Ohio, United States; Benjamin Rose, Cleveland, Ohio, United States; Benjamin Rose Institute on Aging, Cleveland, Ohio, United States; Benjamin Rose, Cleveland, Ohio, United States

## Abstract

Best Program for Caregiving (BPC) is a free, online directory with detailed information on 45 dementia caregiving programs. This easy-to-use database has Professional and Public Versions, making BPC an invaluable resource for both providers and caregivers to access information on proven, evidence-based dementia caregiving programs that meet their needs. The Professional Version was designed for providers to use for identifying and comparing programs, and for making informed decisions on adopting implementation-ready programs. Available data include program characteristics, research outcomes, and delivery organizations’ experiences. The Public Version displays more than 200 organizations verified as delivering one or more of the 45 programs on the Professional Version. By inserting a zip code, providers and family caregivers can find and enroll in locally available programs that align with each caregiver’s individual preferences and care situation. Selected results from the Public Version indicate 85.0% of programs are free; 18 are offered virtually online or by phone to caregivers nationwide; 23.2% were adapted for a diverse group of caregivers such as those living in rural and other underserved communities. Common adaptations include consulting with members or experts of the diverse group (100.0%), adapting program content (92.8%), and delivering the program in the group’s language (53.8%). This presentation provides an overview of programs in and findings from BPC, as well as providing a demonstration and discussion on how providers can use the database as a resource for accessing and implementing evidence-based dementia care at their organization.

